# Eating behavior patterns in relation to obesity phenotypes and beige adipose tissue content with a focus on young women; a narrative review

**DOI:** 10.3389/fnut.2025.1692944

**Published:** 2025-11-04

**Authors:** Katarzyna W. Walkiewicz, Sylwia Dzięgielewska-Gęsiak, Dariusz Myrcik, Martyna Bednarczyk, Małgorzata Muc-Wierzgoń

**Affiliations:** ^1^Department of Internal Disease Propaedeutics and Emergency Medicine, Faculty of Public Health, Medical University of Silesia, Bytom, Poland; ^2^Department of Cancer Prevention, Faculty of Public Health, Medical University of Silesia, Bytom, Poland

**Keywords:** eating styles, phenotypes of obesity, beige adipose tissue, metabolic health, young woman

## Abstract

Eating behaviors extend beyond the physiological satisfaction of hunger; they play a key role in emotion regulation, reward mechanisms, and environmental adaptation. Stable patterns of thoughts, emotions, and food choices create eating styles—complex, individualized behavioral constructs shaped partly by genetic predisposition (e.g., genes influencing food preferences) and partly by family, social, and personality factors. Eating styles are an important determinant of the risk of eating disorders, obesity, and associated metabolic complications. The literature distinguishes adaptive and maladaptive (problematic) styles, which differ in their effects on body weight regulation and health behaviors. Obesity itself is not a homogeneous clinical entity but rather a spectrum of phenotypes differing in adipose tissue distribution (visceral vs. subcutaneous), the severity of metabolic disturbances, and the biological activity of adipocytes. Increasing attention has been paid to the functional diversity of adipose tissue, particularly beige adipose tissue (BeAT), which—through thermogenesis and glucose regulation—is increasingly recognized as a protective factor against insulin resistance and metabolic syndrome. Its activity is highly variable and may be influenced by behavioral factors, including eating patterns. This review aims to summarize current evidence on the relationship between eating styles, obesity phenotypes, and the role of beige adipose tissue. The analysis focuses on young women (<25 years), a group undergoing dynamic hormonal changes and at high risk of establishing persistent eating habits. By integrating psychological and biological determinants of obesity, this paper proposes a conceptual framework linking eating styles, adipose tissue distribution, and metabolic activity—with particular emphasis on BeAT—as a potential target for early prevention of metabolic disorders.

## Introduction

According to expert consensus, obesity can be classified by etiology into primary, secondary, and genetic forms. Primary obesity is the most prevalent type; however, the precise pathomechanism underlying its development has not yet been clearly established. Two major phenotypes of obesity have been described: the android type, characterized by central/visceral fat distribution, and the gynoid type, with adipose tissue predominantly accumulating around the hips and thighs. Central obesity, in particular, is strongly associated with an increased risk of metabolic disorders (1).

The health consequences of excessive adipose tissue accumulation are determined not only by its location but also by its histological and functional characteristics. White adipose tissue (WAT), the principal energy reservoir in the body, is hormonally active and exhibits the most unfavorable metabolic profile (2). In recent years, increasing attention has been paid to other adipocyte subtypes—brown adipose tissue (BAT) and beige adipose tissue (BeAT)—which are capable of interconversion under environmental, hormonal, and dietary influences. Of particular interest is beige adipose tissue, which, through the expression of uncoupling protein 1 (UCP1), expends energy in the form of thermogenesis and may play a protective role against obesity and insulin resistance.

Thus, when addressing the pathophysiology of obesity, it is essential to consider not only fat distribution and anthropometric indices but also the metabolic activity of distinct adipocyte populations. This may explain phenotypic variability among individuals with comparable BMI. For practical purposes, obesity has also been subdivided into clinical and preclinical forms, the latter referring to cases in which abnormal anthropometric indices are not yet accompanied by overt organ dysfunction (1).

In addition to biological mechanisms, problematic eating behaviors—such as emotional, external, or uncontrolled eating—are closely linked to visceral obesity and metabolic disturbances, independent of total caloric intake. These issues appear particularly relevant in young women, where psychosocial factors, hormonal fluctuations, and sociocultural pressures may promote the persistence of maladaptive dietary patterns. Moreover, evidence suggests sex-related differences in beige adipose tissue activity, with higher activity observed in women, which may represent an adaptive mechanism supporting energy homeostasis during the reproductive period.

Young women represent a unique demographic group undergoing dynamic biological and psychosocial transitions. Hormonal fluctuations during adolescence and early adulthood, including variations in estrogen and progesterone, play a critical role in adipose tissue distribution and the regulation of thermogenic activity in beige adipose tissue. Furthermore, endocrine conditions such as polycystic ovary syndrome (PCOS) are highly prevalent in this population, contributing to both metabolic vulnerability and altered eating behaviors. Beyond hormonal factors, young women are also exposed to strong sociocultural pressures regarding body image and eating norms, which increase the risk of disordered eating and unhealthy dietary patterns. Taken together, these intersecting biological and social determinants make young women a particularly relevant group for targeted research on eating behaviors, obesity phenotypes, and beige adipose tissue activity.

**Figure 1:**
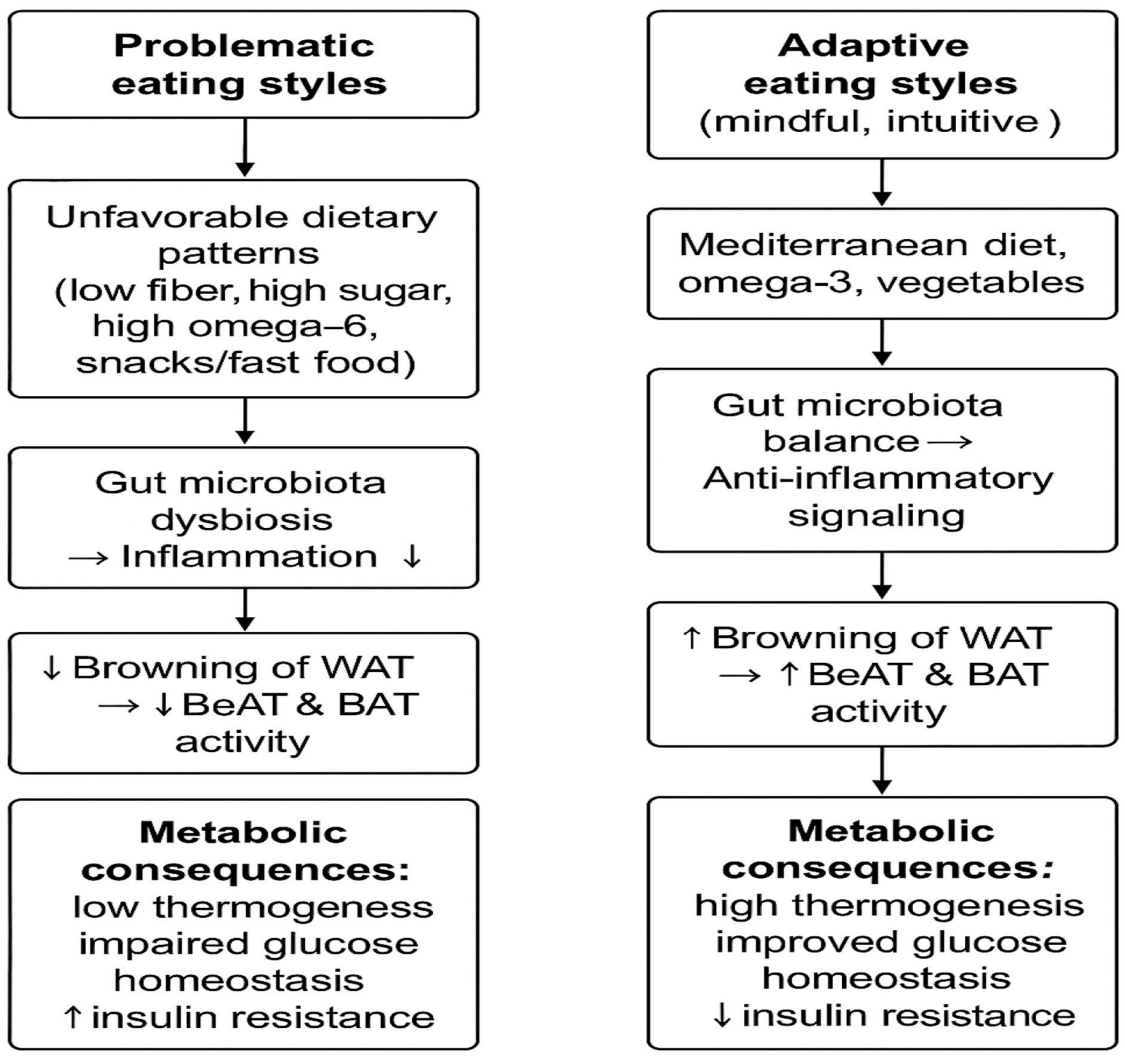
Conceptual framework of the relationship between eating styles, adipose tissue distribution, and beige adipose tissue (BeAT) activity in young women.

Given the importance of early intervention in this age group (18–30 years), the present review focuses on young women, examining the interplay between eating behavior patterns, obesity phenotypes, and the metabolic activity of adipocytes—with particular emphasis on beige adipose tissue as a potential target for the prevention of early metabolic disturbances. The conceptual framework of these relationships is illustrated in Figure 1.

## Eating style and obesity

The relationship between eating behaviors and the development of obesity is often bidirectional. On the one hand, eating habits influence the risk of developing metabolic disorders, but the presence of overweight/obesity, especially during adolescence, is associated with a significantly higher risk of developing abnormal eating patterns and deepening metabolic disorders (3).

Eating styles are generally divided into three subgroups depending on the risk of developing metabolic disorders as a consequence: 1. problematic eating styles (high risk): emotional style, external style, restrictive style, uncontrolled style; 2. optimal eating styles (low risk): intuitive eating, mindful eating; 3. normal eating styles (intermediate risk): focused style, visceral style, sensual style, rhythmic style (4–8). The fundamental difference between problematic and non-problematic eating styles stems from the fact that in problematic styles, internal control over the eating process is disturbed, and nonphysiological stimuli such as emotions, social context become the stimuli for food consumption. It is also worth mentioning that inappropriate eating styles can become a basis for the development of future eating disorders. Their incidence has almost doubled in the last decade, and they currently affect approximately 8.5% of the population (9). This phenomenon primarily affects young women, in whom cultural pressure, hormonal fluctuations, and higher susceptibility to stress may exacerbate problematic eating patterns.

Women are more often diagnosed with eating disorders. This is particularly significant that it is estimated that approximately 30% of women diagnosed with eating disorders during adolescence will develop obesity during their lifetime (10).

Eating styles develop with age, but already at birth, we are predisposed to certain biological predispositions that can influence the development of specific eating patterns, such as a more or less rhythmic mode of satisfying needs or individual sensitivity to sensually stimuli (11). A significant factor shaping eating styles is the family environment, but also the changing external food environment, which has intensified in recent decades. In the modern food environment (highly developed countries), the availability of tasty foods rich in sugar and fat outweighs the rewarding properties of unprocessed foods (12). This promotes overeating, which is the most important behavioral trait associated with obesity and is a component of an uncontrolled and emotional eating style. Emotional eating—in response to states of arousal such as fear, anxiety, anger, or excitement—is particularly associated with the consumption of sweet and energy-rich foods and binge eating. In turn, the easy availability of tasty food—the sight and smell of food—stimulates the perpetuation of an extrinsic eating style, independent of physiological signals of hunger and satiety (13).

In young, obese adults, the most frequently diagnosed eating styles are restrictive and emotional. Furthermore, a more detailed analysis of this group indicates that obese individuals are more likely to overeat driven by negative feelings; consume more processed, sweet, and fried foods; are less likely to eat fresh whole grains, fruits, and vegetables; pay less attention to the sensory and spiritual aspects of eating; are more likely to focus on self-esteem and feeling bad about overeating; are more likely to eat in a hectic, tense atmosphere; are more likely to eat while doing other things, and are more likely to eat alone (14). Furthermore, it has been shown that different eating styles may explain the varying incidence of obesity, despite similar genetic predisposition. In the Finnish study, the genetic background was taken into account, limiting the analysis to 39 pairs of monozygotic female twins and 45 pairs of male twins discordant in terms of obesity or overweight; in these cases, a problematic: restrictive, emotional, or uncontrolled eating style was statistically significantly more often associated with the occurrence of excess body weight and compensated for the lack of differences in the genetic material of the subjects (15).

Based on available analyses, it is difficult to clearly determine how the diet composition of young obese individuals differs from that of their healthy peers, but there is also ample evidence that their eating behaviors are altered (16). It appears that in girls and young women, eating habits and lifestyle during adolescence are particularly important in shaping their metabolic phenotype and susceptibility to obesity.

An analysis by Polish researchers showed that snacking is a major factor contributing to unhealthy fat consumption among school-age children, especially those with excess body weight. In this group, the average fat intake from snacks covered almost 47% of the recommended daily intake. Over 12% of students exceeded the recommendations for total fat, 20% exceeded the SFA (saturated fatty acid) intake limits, and over 30% exceeded the TFA (trans fat) intake threshold. Girls had a slightly higher intake of total fat and SFA than boys, and total fat intake was highest among overweight children (17). Another study confirmed the existence of a relationship: greater sensitivity to food = increased sensitivity to sensory stimuli = > presence of anthropometric markers of abdominal obesity. Stratified analysis confirmed that girls aged 9–14 years tend to have higher total sugar consumption than boys, and trends established in childhood may persist into older age and lead to metabolic health consequences (18). It was also confirmed for the Asian population that school-age girls consume more sugar than boys (19). Interestingly, another analysis showed that overweight young people also tend to frequently choose “light” snacks (20).

It is also noting a key role of chronotype of food intake, which plays a role in the development of obesity. In van der Merwe’s study, despite finding no differences in the quantity and quality of food consumed across the study groups, differences in the incidence of overweight and obesity were observed depending on the timing of meal consumption. Individuals with a late evening chronotype (ET)—most calories consumed after 6 p.m. and at night—were more likely to be clinically overweight and obese than those with an early morning chronotype (MT). Both chronotypes in the study had similar energy and macronutrient intakes (21).

Finally, it seems worthwhile to highlight the role of biological factors, such as prenatal exposure to sex hormones and the hormonal balance of adult women, in shaping eating habits. Gębala’s study demonstrated that optimal physical activity and adequate prenatal exposure to female sex hormones have a beneficial effect on maintaining normal anthropometric parameters and proper fat tissue distribution in young adult. Women with indirectly confirmed optimal prenatal exposure to sex hormones were more likely to engage in physical activity and better control their body weight in adult (22). The impact of hormonal imbalances on eating habits and, consequently, the development of metabolic disorders was also analyzed in a study comparing young women diagnosed with polycystic ovary syndrome (PCOS) with healthy nulliparous women. PCOS is one of the best-documented endocrine metabolic disorders in women of reproductive age, and its clinical picture often coexists with abnormal eating patterns and abdominal obesity. This study revealed that patients with PCOS were slightly more likely to exhibit emotional eating characteristics, but the differences were not statistically significant (*p* = 0.11). Similarly, quality of life scores (physical and mental) did not differ between women with and without PCOS. These results suggest that infertile women with PCOS and obesity and infertile women with obesity without PCOS do not have different eating habits and are characterized by similar mental and physical quality of life. An interesting field of further research would be to compare these characteristics with a group of young mothers (23).

## Biological activity of beige adipose tissue

There are three histological types of adipose tissue: white adipocytes (WAT), beige, and brown (BAT) (2). Their main characteristics are summarized in Table 1.

**Table 1:** Histological and functional characteristics of white, brown, and beige adipose tissue.

Problematic eating styles	Adaptive eating styles (mindful, intuitive)
Unfavorable eating styles (low fiber, high sugar, high omega-6, snacks/fast food)	Mediterranean dieta, omega-3, vegetables
Gut microbiota dysbiosis➔ Inflammation	Gut microbiota balance➔ anti-inflammatory signaling
↓Browning of WAT➔↓BeAT&BAT activity	↑Browning of WAT➔ ↑BeAT&BAT activity
*Metabolic consequences* Low thermogenesis, impaired glucose homeostasis, ↑insulin resistance	*Metabolic consequences* High thermogenesis, improve glucose homeostasis, ↓insulin resistance

WAT is composed primarily of unilocular adipocytes, which accumulate triglycerides in the cytosol. In response to hormonal stimuli (insulin, catecholamines, and glucagon), white adipocytes can store or mobilize energy substrates into the circulatory system to maintain the body’s energy homeostasis (2).

In contrast, beige adipocytes are characterized by a rich mitochondrial content and significant expression of uncoupling protein type 1 (UCP1), responsible for thermogenesis. White and brown adipocytes originate from different precursor cells, but in the adult, processes leading to the mutual transformation of white adipocytes into brown adipocytes and vice versa may be activated (24). Therefore, morphologically and functionally, beige adipocytes are an intermediate stage between their white and brown counterparts, and their formation (“browning” of adipose tissue) is induced by various biological and environmental factors and represents a metabolically beneficial process of white adipose tissue remodeling. Beige adipocytes contribute to energy expenditure by thermoregulation through the activity of the uncoupling protein UCP1 (25, 26). Data suggest that the activity of beige and brown adipose tissue may be higher in women than in men, which is associated with higher estrogen levels, a different receptor profile, and greater sensitivity to environmental stimuli such as cold. This phenomenon may represent an adaptive metabolic mechanism in young women. Factors that may influence the transformation of white adipocytes into beige are endogenous and exogenous, and include: reduced calorie intake (fasting, including various forms of intermittent fasting), a diet rich in antioxidants, physical activity, stress, modulation of the gastrointestinal microbiota, exposure to low temperature, hormonal activity (catecholamines, thyroid hormones, estrogenes), and the paracrine effect of substances secreted by beige and brown adipocytes (batokines) (27–31). It is also worth mentioning a similar effect that may be initiated by the use of certain medications (metformin, GLP-1 analogs, thiazolidinediones), thus constituting an additional premise for implementing such therapy in obese patients (32). Similarly, in the development of obesity, we also observe a number of changes that can lead to the reverse process, namely the “debrowning” of adipose tissue and impaired thermogenesis. Experimental studies have noted that „debrowning” is one of the earliest changes occurring in adipose tissue in obese patients. This process includes, among others, a decrease in the level of angiogenesis markers, an increase in inflammatory cytokine activity, and the activation of oxidative stress pathways (29, 33).

Patients with BMI > 35 kg/m^2^ (second stage of obesity) exhibit significant deficiencies in beige and brown adipose tissue. In Herz’s analysis, active beige adipose tissue was detected in only 35% of the individuals on 18F-FDG PET. A significantly higher incidence of cold-induced thermogenesis was observed in this group. Furthermore, obese patients with active BAT had a 28.8% lower visceral fat mass, despite a slightly higher total fat mass, compared to individuals without detectable 18F-FDG uptake by BAT. In turn, a lower percentage of visceral adipose tissue was associated with lower insulin resistance and lower markers of systemic inflammation (34).

## The impact of diet on browning adipose tissue

The above data indicate a metabolically beneficial effect of adipose tissue browning. Changing dietary habits, including modifying eating habits, may be a significant intervention leading to the replacement of white adipocytes with beige ones and the activation of induced thermogenesis mechanisms. Dietary interventions that may influence adipocyte browning include supplementing the diet with capsaicin-rich foods, fresh vegetables, and fruits, supplementing omega-3 polyunsaturated fatty acids and maintaining an appropriate omega-3 to omega-6 ratio, calorie restriction, and intermittent fasting (35). The main dietary factors associated with the induction or inhibition of adipose tissue browning are summarized in Table 2.

**Table 2:** Dietary factors influencing browning and debrowning of adipose tissue.

Dietary factor/pattern	Proposed effect on adipose tissue	Mechanism of action	References
Capsaicin (chili peppers, spicy foods)	↑ Browning	Activation of TRPV1, stimulation of UCP1 expression	(35)
Fruits and vegetables (polyphenols, fiber)	↑ Browning	Antioxidant activity, AMPK–PGC1α pathway	(35)
Omega-3 fatty acids (EPA, DHA)	↑ Browning	Mitochondrial biogenesis, anti-inflammatory mediators	(35)
Balanced omega-3/omega-6 ratio	↑ Browning/↓ Debrowning	Competitive metabolism; anti-inflammatory signaling	(38)
Calorie restriction/intermittent fasting	↑ Browning	Increased fatty acid oxidation, hormonal modulation	(35)
Excessive omega-6 intake	↓ Browning (↑ Debrowning)	Pro-inflammatory eicosanoids, oxidative stress	(37, 38)
Diet rich in snacks, fast food, low fiber	↓ Browning (↑ Debrowning)	Nutrient imbalance, inflammation, impaired microbiota	(36)

Research indicates that in individuals with problematic eating styles, particularly emotional eating and uncontrolled eating, the supply of fresh vegetables and products rich in omega-3 fatty acids is low, and the preferred products are sweet and salty snacks, fast food, high sodium products, and low fiber (36). Furthermore, in individuals with compulsive behaviors, including binge eating episodes, there is an imbalance in the supply of unsaturated omega-3/omega-6 fatty acids, with an increased supply of omega-6, which in this context may contribute to the induction of inflammatory processes and the “debrowning” of adipose tissue (37). Under physiological conditions, omega-3 and omega-6 acids are metabolized competitively by the same set of enzymes; but lipid mediators produced as a result of their metabolism perform opposing functions. Those derived from omega-6 fatty acids increase inflammation, platelet aggregation, and vasoconstriction. Excessive consumption of omega-6 polyunsaturated fatty acids with a reduced supply of omega-3 polyunsaturated fatty acids is strongly associated with the pathogenesis of adipose tissue dysfunction (38).

In addition, in an animal study, a high-fat diet was found to inhibit the browning of WAT by inhibiting the expression of PGC-1α-IRISIN-UCP-1 in adipose tissue and skeletal muscle. It was also found that this effect could be reversed by increasing physical activity and modifying the diet toward an energy deficit, noting that the effects of the combined intervention were better than those of either intervention alone. Eating large amounts of high-fat foods is characteristic of problematic eating styles, especially emotional and uncontrolled eating (39). This observations were confirmed in another study, where confirming also, that a high-fat diet also impairs the thermogenic function of BAT, and these effects can be minimized i.a. by irisin supplementation. It is important, that the beneficial effect of irisin gradually disappears with the development of clinically apparent obesity and other markers of metabolic syndrome, which may contribute to the “irisin resistance” (analogous to insulin resistance); this phenomenon particularly affected in patients with visceral fat distribution, high serum LDL cholesterol and triglyceride levels, and poorly controlled type 2 diabetes. Pathological eating habits, among other factors, may contribute to the development of these metabolic disorders (40, 41).

## Gut microbiome and beige adipose tissue modulation

The influence of gut microbiota composition on BAT activation and metabolism has been demonstrated in mouse models (42, 43) whereas evidence from human studies remains limited (44).

The gut microbiome has emerged as an important regulator of adipose tissue browning, acting through multiple metabolic and immunological pathways. Microbial-derived molecules influence thermogenic capacity not only by shaping mitochondrial function but also by modifying systemic signaling cascades that support energy dissipation.

Cellular and molecular mechanisms: One of the principal pathways involves bile acids, which, through activation of FXR and TGR5 receptors, stimulate UCP1 expression and enhance thermogenic activity (35, 45). Short-chain fatty acids (SCFAs) such as acetate, butyrate, and lactate represent another key group of microbial metabolites. They promote mitochondrial biogenesis and upregulate UCP1 expression, facilitating the conversion of white adipocytes into a beige phenotype (46, 47). In addition, interactions between gut microbiota and the host immune system can modulate low-grade inflammation, creating a microenvironment favorable for browning (45). Recent reviews further emphasize that microbiota-driven regulation of mitochondrial activity and inflammatory pathways provides an integrative framework that links energy metabolism with immune homeostasis (48, 49).

The principal mechanisms through which the gut microbiota influences adipose tissue browning are summarized in Table 3. This table summarizes current evidence on the role of the gut microbiota in BeAT activation and thermogenesis. Most mechanistic insights derive from animal models, while human data remain limited and require further confirmation in clinical studies.

**Table 3:** Evidence on gut microbiota and beige adipose tissue (BeAT) modulation from animal and human studies.

Model	Mechanism/Mediator	Key findings	References
Animal studies	Short-chain fatty acids (acetate, butyrate, lactate)	Promote mitochondrial biogenesis and UCP1 induction → browning of white adipose tissue	(39, 46, 47)
	Bile acids (FXR/TGR5 signaling)	Stimulate UCP1 expression, enhance thermogenesis	(35, 45)
	Microbiota depletion / transplantation (intermittent fasting, EODF)	Depletion prevents browning; transplantation restores thermogenic response	(47)
	Exercise-induced microbiota shifts	Increased SCFA production, improved browning potential	(50)
	Probiotics	Suggested role in browning; evidence inconsistent	(35)
Human studies	Gut microbiota composition vs. BAT activity (18F-FDG PET, young adults, mean age ~21 y)	↑ Bifidobacterium associated with higher BAT activity; ↑ Akkermansia, Lachnospiraceae, Ruminococcus associated with lower BAT activity	(44)
	Lifestyle factors (dietary shifts, intermittent fasting, physical activity)	Indirect associations via microbiota composition and SCFA production; limited clinical data	(44, 50)

Lifestyle and experimental interventions: Several external factors have been shown to shape microbiota-related browning. Intermittent fasting, particularly the alternate-day fasting regimen (EODF), induces remodeling of the gut microbiota, increases SCFA production, and promotes beige adipocyte activation. Notably, in animal models, microbiota-depleted mice were resistant to fasting-induced browning, whereas transplantation of microbiota from EODF-treated animals restored the thermogenic response (49). Physical activity represents another modifiable factor, enhancing SCFA production and improving glucose homeostasis, thereby indirectly supporting beige fat activation (50). Probiotic supplementation, although biologically plausible, currently lacks consistent evidence for a direct effect on adipose tissue browning (35).

Human evidence: Data from human studies, though limited, begin to confirm these experimental observations. In a cohort of young adults (mean age ~21.8 years, predominantly women), gut microbiota composition was directly associated with brown adipose tissue activity. Specifically, a higher abundance of *Bifidobacterium* correlated positively with BAT volume and activity, while *Akkermansia*, *Lachnospiraceae*, and *Ruminococcus* showed negative associations (44). These findings suggest that interindividual variability in microbiota composition may partly explain differences in thermogenic capacity among young women.

Implications for young women: Considering the metabolic vulnerability of young women—particularly those with hormonal imbalances such as polycystic ovary syndrome (PCOS), eating disorders, or a predisposition to abdominal obesity—microbiota-targeted interventions may represent a promising preventive strategy. Approaches including intermittent fasting, SCFA-promoting dietary patterns, or structured physical activity not only improve metabolic health but also beneficially modulate the gut microbiota, thereby enhancing the potential for beige adipose tissue activation and thermogenic flexibility.

## Limitations

Although some of the studies cited in this review included both male and female participants, the interpretation and conclusions were focused on young women, given their unique hormonal, psychosocial, and metabolic characteristics.

## Conclusion

Eating behavior patterns in young women are a key and multifaceted determinant of obesity phenotypes and their associated metabolic outcomes. Problematic eating styles—particularly emotional, external, and uncontrolled eating—favor visceral fat accumulation and may aggravate metabolic disturbances, independently of genetic predisposition.

Concurrently, beige adipose tissue (BeAT) has emerged as a metabolically active component that may counterbalance the adverse effects of excessive white adipose tissue, especially in young women. Its activity is shaped by behavioral factors (dietary patterns, circadian timing of meals), as well as by hormonal and environmental influences.

Elucidating the interplay between eating behaviors, adipose tissue distribution, and adipocyte metabolic activity provides a foundation for early, individualized obesity-prevention strategies. Targeted nutritional interventions—focusing on the modification of eating styles, improvement of dietary quality, optimization of omega-3/omega-6 fatty acid balance, and promotion of a favorable gut microbiota profile—may stimulate BeAT activity and support metabolic health in young adulthood. Further research is needed to establish effective translational strategies in this field.
